# Analyzing network pharmacology and molecular docking to clarify Duhuo Jisheng decoction potential mechanism of osteoarthritis mitigation

**DOI:** 10.1097/MD.0000000000032132

**Published:** 2022-12-16

**Authors:** Zhenhai Cui, Weidong Zhang, Xuezhen Le, Kunyu Song, Chunliang Zhang, Wenhai Zhao, Liquan Sha

**Affiliations:** a Changchun University of Chinese Medicine, Changchun, Jilin, China; b The Third Affiliated Hospital of the Changchun University of Chinese Medicine, Changchun, Jilin, China; c Affiliated Hospital of the Changchun University of Chinese Medicine, Changchun, Jilin, China.

**Keywords:** Duhuo Jisheng decoction, molecular docking, network pharmacology, osteoarthritis

## Abstract

As a classic remedy for treating Osteoarthritis (OA), Duhuo Jisheng decoction has successfully treated countless patients. Nevertheless, its specific mechanism is unknown. This study explored the active constituents of Duhuo Jisheng decoction and the potential molecular mechanisms for treating OA using a Network Pharmacology approaches. Screening active components and corresponding targets of Duhuo parasite decoction by traditional Chinese medicine systems pharmacology database and analysis platform database. Combining the following databases yielded OA disease targets: GeneCards, DrugBank, PharmGkb, Online Mendelian Inheritance in Man, and therapeutic target database. The interaction analysis of the herb-active ingredient-core target network and protein–protein interaction protein network was constructed by STRING platform and Cytoscape software. Gene ontology functional enrichment analysis and Kyoto Encyclopedia of Genes and Genomes pathway enrichment analysis were carried out. PyMOL and other software were used to verify the molecular docking between the essential active components and the core target. 262 active ingredients were screened, and their main components were quercetin, kaempferol, wogonin, baicalein, and beta-carotene. 108 intersection targets of disease and drug were identified, and their main components were RELA, FOS, STAT3, MAPK14, MAPK1, JUN, and ESR1. Gene ontology analysis showed that the key targets were mainly involved in biological processes such as response to lipopolysaccharide, response to xenobiotic stimulus, and response to nutrient levels. The results of Kyoto Encyclopedia of Genes and Genomes analysis show that the signal pathways include the AGE − RAGE signaling pathway, IL − 17 signaling pathway, TNF signaling pathway, and Toll − like receptor signaling pathway. Molecular docking showed that the main active components of Duhuo parasitic decoction had a good bonding activity with the key targets in treating OA. Duhuo Jisheng decoction can reduce the immune-inflammatory reaction, inhibit apoptosis of chondrocytes, strengthen proliferation and repair of chondrocytes and reduce the inflammatory response in a multi-component-multi-target-multi-pathway way to play a role in the treatment of OA.

## 1. Introduction

Osteoarthritis (OA) is a disease where the articular cartilage degenerates and joints ache. Its pathological features include articular cartilage degradation, synovitis, osteophyte formation, subchondral bone remodeling, and other joint structural changes.^[1]^ Statistics show that 18.6% of women and 9.6% of men over 60 have OA, and 25% cannot perform daily activities. It is estimated that there will be 130 million OA patients by 2050.^[2,3]^ With age, cartilage and subchondral bone metabolism are abnormal, and the joint will appear with weird biomechanical imbalance.^[4]^ There are many ways to treat OA clinically. It is found that Duhuo Jisheng decoction has the advantages of remarkable curative effect, fewer adverse reactions, and low cost in OA treatment.

Duhuo Jisheng decoction is a classical prescription of Prepare for an urgent and essential medication written by Simiao Sun, composed of Radix Angelicae Biseratae (Duhuo), Herba Taxilli (Sangjisheng), Chuanxiong Rhizoma (Chuanxiong), Angelicae Sinensis Radix (Danggui), Eucommiae Cortex (Duzhong), Saposhnikoviae Radix (Fangfeng), Poria Cocos(Schw.) Wolf. (Fuling), licorice (Gancao), Achyranthis Bidentatae Radix (Niuxi), Paeoniae Radix Alba (Baishao), Gentiana Macrophylla Pall (Qinjiao), Asari Radix Et Rhizoma (Xixin), Panax Ginseng C. A. Mey. (Renshen), Cinnanmomi Cortex (Rougui), and Rehmanniae Radix Praeparata (Dihuang) dried root. It has the effect of dispelling rheumatism, stopping arthralgia, benefiting the liver and kidney, tonifying qi and blood, and relieving OA pain and other symptoms. According to the literature research, it is found that the treatment of OA with Duhuo Jisheng decoction is realized by regulating a variety of cytokines, signal pathways, and gene expression.^[5–8]^ Therefore, exploring the molecular mechanism of Duhuo Jisheng decoction in OA treatment has significant scientific value and social significance.

Based on constructing the interaction network of “drug-component-target-disease,” network pharmacology uses the method of system biology and database analysis to screen out actual research results. And systematically study the network intervention mode and influence of drugs on disease from the complex network relationship.^[9]^ Based on the lock-key model, molecular docking is a method for virtual screening drug targets and predicting active components by simulating the interaction between ligands and receptors and predicting the binding mode and intensity of ligands and receptors based on molecular principles.^[10]^ The purpose of this study is to explore the potential mechanisms of Duhuo Jisheng decoction and provide a reference for further research and clinical application of the remedy.

## 2. Methods

### 2.1. Screening of active components

By accessing the traditional Chinese medicine systems pharmacology database and analysis platform (TCMSP) database, taking Radix Angelicae Biseratae (Duhuo), Herba Taxilli (Sangjisheng), Chuanxiong Rhizoma (Chuanxiong), Angelicae Sinensis Radix (Danggui), Eucommiae Cortex (Duzhong), Saposhnikoviae Radix (Fangfeng), Poria Cocos(Schw.) Wolf. (Fuling), licorice (Gancao), Achyranthis Bidentatae Radix (Niuxi), Paeoniae Radix Alba (Baishao), Gentiana Macrophylla Pall (Qinjiao), Asari Radix Et Rhizoma (Xixin), Panax Ginseng C. A. Mey. (Renshen), Cinnanmomi Cortex (Rougui), and Rehmanniae Radix Praeparata (Dihuang) dried root. Like keywords, the information of the above active ingredients was screened in the database. The screening criteria of oral bioavailability ≥ 30% and drug similarity (Drug-like ≥ 0.18) were set to establish the collection of active components of Duhuo Jisheng decoction.

### 2.2. Prediction of targets of active components

The TCMSP database queried the target protein of each functional element of Duhuo Jisheng decoction, and the target of all the active ingredients collected was predicted by Perl software. The UniProt database selected the species as “Homo Sapiens” for retrieval, and each target protein’s corresponding target gene names were obtained. Then the target gene name collection was established, and the duplicate genes were deleted. The result is the target of the active components of Duhuo Jisheng decoction.

### 2.3. Prediction of OA targets

Use “OA” as the keyword to search the relevant targets in GeneCards, DrugBank, PharmGkb, Online Mendelian Inheritance in Man, and therapeutic target database databases, respectively, then integrate the search results of 5 databases and delete duplicates. The final target is the related target of OA.

### 2.4. Prediction of potential targets of compound therapy for OA

The microarray packet VennDiagram package in R (version 4.1.3) software was used to screen the common targets of diseases and drugs.

### 2.5. Network construction of “herb-active ingredient-action target”

The active components and core targets of Duhuo Jisheng decoction were analyzed by STRING11.5 platform and Cytoscape3.8.0 software for protein–protein interaction (PPI) protein network interaction. Then the regulatory network was constructed by using the NetworkAnalyze plug-in in Cytoscape to form the “herb-active ingredient-action target” network diagram of Duhuo Jisheng decoction in the treatment of OA.

### 2.6. PPI network construction and network topology analysis

Input the potential target of Duhuo Jisheng decoction in the treatment of OA into the STRING11.5 platform, and select the species as “HomoSapiens” to obtain the protein interaction relationship. The lowest interaction threshold is set to > 0.9. Hide the discrete point, and other parameters remain at the default setting to construct the PPI network model. The CytoNCA plug-in in Cytoscape3.8.0 software was used to screen the highly enriched protein interactions and build the PPI core network map.

### 2.7. Enrichment analysis of gene ontology (GO) and Kyoto Encyclopedia of Genes and Genomes (KEGG) pathways

Based on the R packet cluster profile 4.2.1 of Bioconductor, the core target was analyzed by GO functional enrichment analysis (gene ontology) and KEGG pathway analysis (KEGG pathway analysis), and the main biological functions and main signal pathways of Duhuo Jisheng decoction in the treatment of OA were studied. The species was selected as “Homo Sapiens,” and the threshold was set at *P* < .05. The bubble chart of GO enrichment analysis and the column chart of KEGG enrichment analysis was made.

### 2.8. Molecular docking

To further explore the regulatory effect of Duhuo Jisheng decoction on OA, the main chemical constituents were docked with the selected core targets. The 3D structure of crucial compounds was made by Chem Office software, and then the 3D design of the core protein gene was downloaded from the PDB database. The water molecules and small molecular ligands of protein structure were deleted by PyMol software and introduced into Auto Dock Tools for hydrogenation and other pretreatments. Convert the active ingredient and target protein into a pdbqt format file, and look for the functional pocket. Finally, Auto Dock Tools were run to dock the active components and target proteins, respectively, and the lowest binding energy data was saved due to molecular docking. Then the critical activity of the 2 is evaluated according to the binding energy, and the binding energy ≤ −5.0 kJ/mol is selected as the screening basis to assess the possibility of network analysis and prediction.

### 2.9. The article deals with databases

See Table 1.

**Table 1:** Database information involved in the article.

No.	Database name	Web site
1	TCMSP	https://old.tcmsp-e.com/tcmsp.php
2	UniProt	https://www.uniprot.org/
3	GeneCardsda	https://www.genecards.org/
4	DrugBank	https://www.drugbank.ca/
5	PharmGkb	https://www.pharmgkb.org/
6	OMIM	https://omim.org/
7	TTD	http://db.idrblab.net/ttd/
9	PDB	http://www.rcsb.org/

## 3. Results

### 3.1. Screening of active components and prediction of targets

A total of 262 active chemical constituents and 4257 predicted targets of Duhuo Jisheng decoction were obtained by searching the TCMSP database. Statistics showed that there were 9 from Radix Angelicae Biseratae (Duhuo), 2 from Herba Taxilli (Sangjisheng), 7 from Chuanxiong Rhizoma (Chuanxiong), 2 from Angelicae Sinensis Radix (Danggui), 28 from Eucommiae Cortex (Duzhong), 18 from Saposhnikoviae Radix (Fangfeng), 15 from Poria Cocos(Schw.) Wolf. (Fuling), 92 from licorice (Gancao), 20 from Achyranthis Bidentatae Radix (Niuxi), 20 from Paeoniae Radix Alba (Baishao), 2 from Gentiana Macrophylla Pall (Qinjiao), 8 from Asari Radix Et Rhizoma (Xixin), 22 from Panax Ginseng C. A. Mey. (Renshen), 15 from Cinnanmomi Cortex (Rougui), and 2 from Rehmanniae Radix Praeparata (Dihuang), The active ingredients are shown in Table 2.

**Table 2:** Information about the active ingredients in Duhuo Jisheng decoction.

Mol ID	Molecule Name	OB	DL	Herb name
MOL001941	Ammidin	34.55	0.22	Radix Angelicae Biseratae
MOL001942	isoimperatorin	45.46	0.23	Radix Angelicae Biseratae
MOL000358	beta-sitosterol	36.91	0.75	Radix Angelicae Biseratae
MOL000359	sitosterol	36.91	0.75	Herba Taxilli
MOL000098	quercetin	46.43	0.28	Herba Taxilli
MOL002135	Myricanone	40.6	0.51	Chuanxiong Rhizoma
MOL002140	Perlolyrine	65.95	0.27	Chuanxiong Rhizoma
MOL000358	beta-sitosterol	36.91	0.75	Angelicae Sinensis Radix
MOL000449	Stigmasterol	43.83	0.76	Angelicae Sinensis Radix
MOL002058	40957-99-1	57.2	0.62	Eucommiae Cortex
MOL000211	Mairin	55.38	0.78	Eucommiae Cortex
MOL000358	beta-sitosterol	36.91	0.75	Eucommiae Cortex
MOL006709	AIDS214634	92.43	0.55	Eucommiae Cortex
MOL011732	anomalin	59.65	0.66	Saposhnikoviae Radix
MOL011737	divaricatacid	87	0.32	Saposhnikoviae Radix
MOL011740	divaricatol	31.65	0.38	Saposhnikoviae Radix
MOL001941	Ammidin	34.55	0.22	Saposhnikoviae Radix
MOL000275	trametenolic acid	38.71	0.8	Poria Cocos(Schw.) Wolf.
MOL000279	Cerevisterol	37.96	0.77	Poria Cocos(Schw.) Wolf.
MOL001484	Inermine	75.18	0.54	licorice
MOL001792	DFV	32.76	0.18	licorice
MOL000211	Mairin	55.38	0.78	licorice
MOL002311	Glycyrol	90.78	0.67	licorice
MOL012461	28-norolean-17-en-3-ol	35.93	0.78	Achyranthis Bidentatae Radix
MOL012505	bidentatoside,ii_qt	31.76	0.59	Achyranthis Bidentatae Radix
MOL012537	Spinoside A	41.75	0.4	Achyranthis Bidentatae Radix
MOL000211	Mairin	55.38	0.78	Paeoniae Radix Alba
MOL000358	beta-sitosterol	36.91	0.75	Paeoniae Radix Alba
MOL000359	sitosterol	36.91	0.75	Paeoniae Radix Alba
MOL000422	kaempferol	41.88	0.24	Paeoniae Radix Alba
MOL000358	beta-sitosterol	36.91	0.75	Gentiana Macrophylla Pall
MOL000359	sitosterol	36.91	0.75	Gentiana Macrophylla Pall
MOL002879	Diop	43.59	0.39	Panax Ginseng C. A. Mey.
MOL000449	Stigmasterol	43.83	0.76	Panax Ginseng C. A. Mey.
MOL000358	beta-sitosterol	36.91	0.75	Panax Ginseng C. A. Mey.
MOL000131	EIC	41.9	0.14	Cinnanmomi Cortex
MOL000359	sitosterol	36.91	0.75	Rehmanniae Radix Praeparata
MOL000449	Stigmasterol	43.83	0.76	Rehmanniae Radix Praeparata
MOL012141	Caribine	37.06	0.83	Asari Radix Et Rhizoma
MOL001460	Cryptopin	78.74	0.72	Asari Radix Et Rhizoma
MOL001558	sesamin	56.55	0.83	Asari Radix Et Rhizoma

### 3.2. Prediction results of potential targets of compound therapy for OA

**Figure 1.**
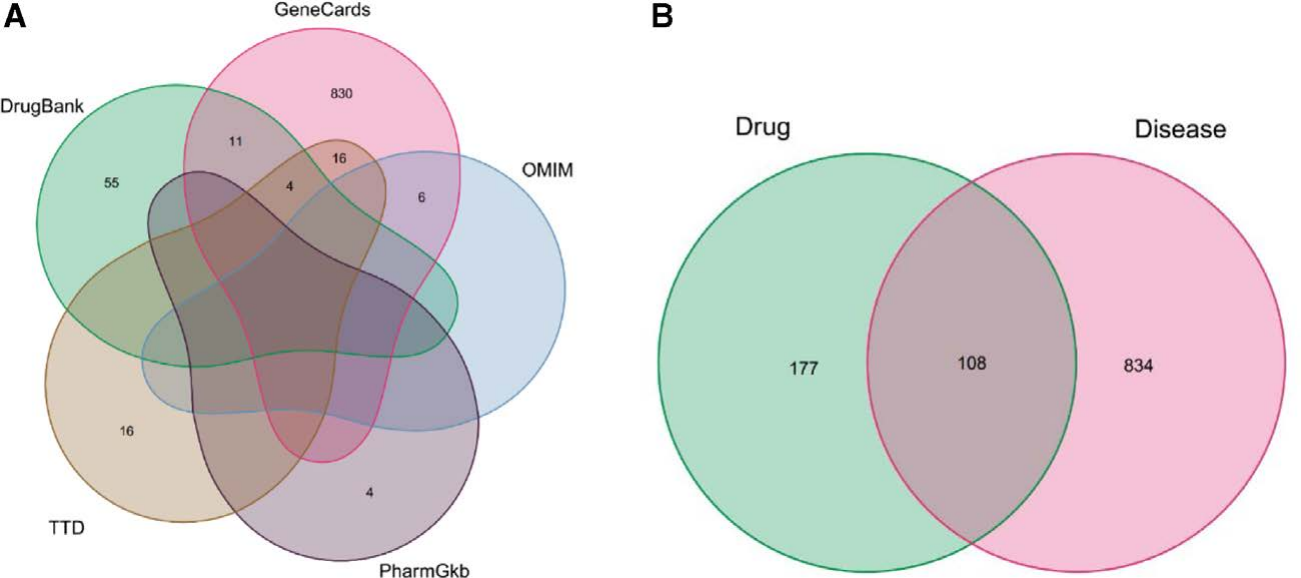
Venn diagram.

For OA, GeneCards provided 3262 disease targets, DrugBank provided 48, PharmGKB provided 4, Online Mendelian Inheritance in Man provided 6, and therapeutic target database provided 38. Following the summarization and deletion of duplicates, 4358 arthritis-related targets were found. See Figure 1A. In the next step, the data of drug targets and disease targets were imported into R software, and the intersection genes were obtained using Draw Venn Diagrams. We drew a Venn diagram, and 108 intersection target genes were identified. See Figure 1B.

### 3.3. The network diagram of “herb-active ingredient-action target”

**Figure 2.**
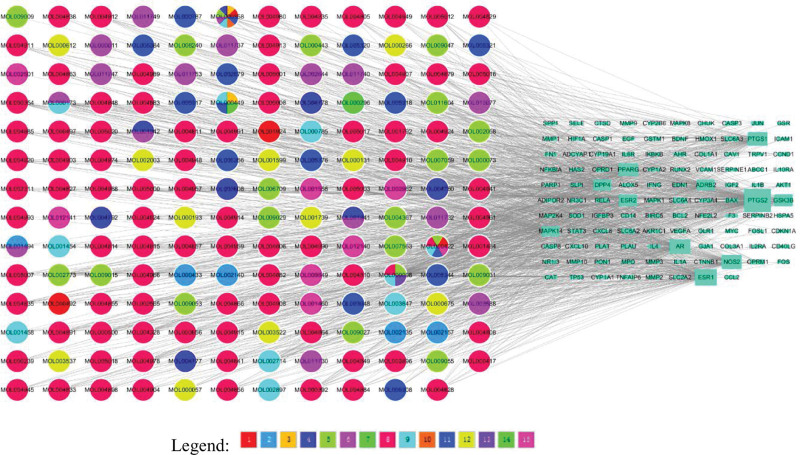
The “herb-active ingredient-action target” network diagram.

The “herb-active ingredient-action target” network diagram was constructed using the Network Analyze plug-in in Cytoscape 3.8.0 software. The network diagram shows that there are 275 nodes and 1116 edges. Different colors represent different drugs, and each edge represents the relationship between the compound and the target (Fig. 2). By counting the degrees of the actives, we can determine how critical the compounds are in the network. The top 5 actives are MOL000098 (quercetin, degree = 71), MOL000422 (kaempferol, degree = 30), MOL000173 (wogonin,degree = 25), MOL002714 (baicalein, degree = 18), MOL002773(β-beta-carotene, degree = 18) (Table 3).

**Table 3:**
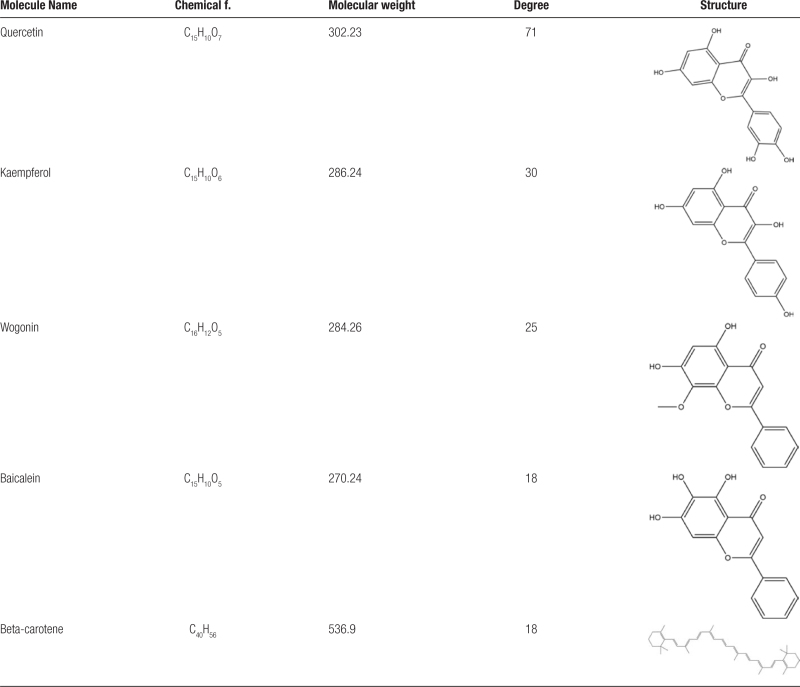
Basic information of key compounds.

### 3.4. Results of PPI network and network topology analysis

**Figure 3.**
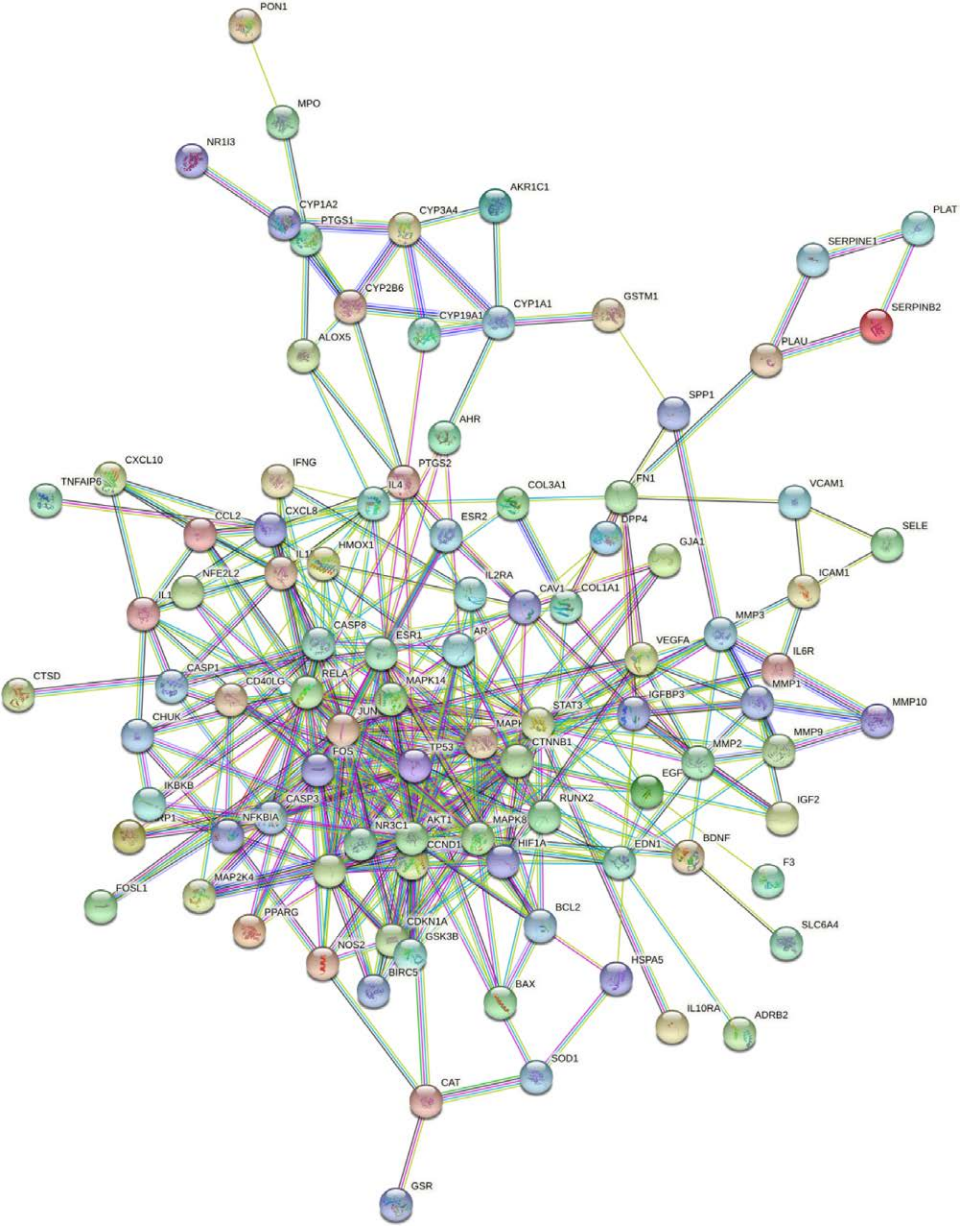
The PPI network diagram. PPI = protein–protein interaction.

**Figure 4.**
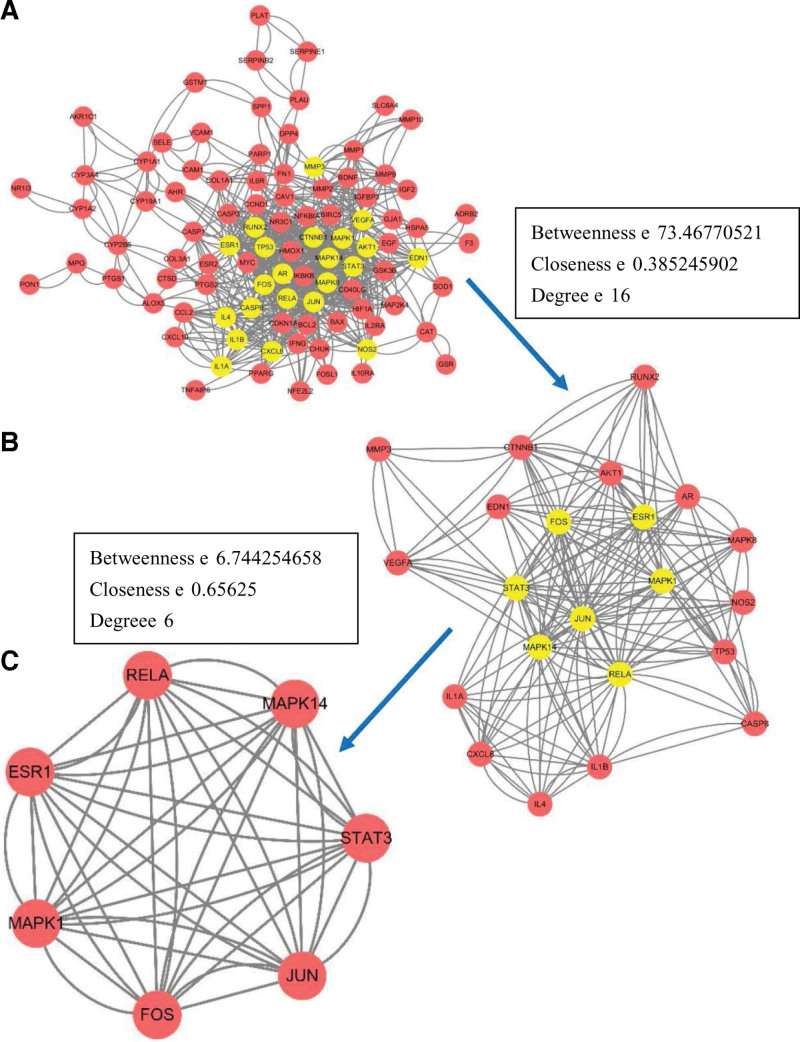
PPI network diagram of key targets. PPI = protein–protein interaction.

The STRING11.5 platform was used to construct the protein interaction network of the overlapping genes. The network diagram consists of 108 nodes and 370 edges. The node represents the protein, and the edge represents the interaction between protein and protein. The higher the degree of network connection, the closer the relationship between proteins (Fig. 3).To further screen the core protein, the interaction.tsv file obtained by the STRING11.5 platform is imported into Cytoscape, and the CytoNCA plug-in analyzes the network. Firstly, Betweenness ≥ 73.46770521, Closeness ≥ 0.385245902, and Degree ≥ 16 are screening conditions. A central network diagram containing 95 nodes and 740 edges is constructed (Fig. 4A). Subsequently, the screening conditions were set as Betweenness ≥ 6.744254658, Closeness ≥ 0.65625, and Degree ≥ 6. A central network diagram with 22 nodes and 190 edges is constructed (Fig. 4B). The darker the color and the closer to the center indicates that the RANK is earlier and more critical. The network diagram shows that the network is the most connected: RELA, FOS, STAT3, MAPK14, MAPK1, JUN, and ESR1 (Fig. 4C). It is suggested that the corresponding targets of these protein genes play an essential role in treating OA with Duhuo parasitic decoction and may be the critical target of Duhuo Jisheng decoction in the treatment of OA.

### 3.5. Enrichment analysis of GO and KEGG pathways of overlapping genes

**Figure 5.**
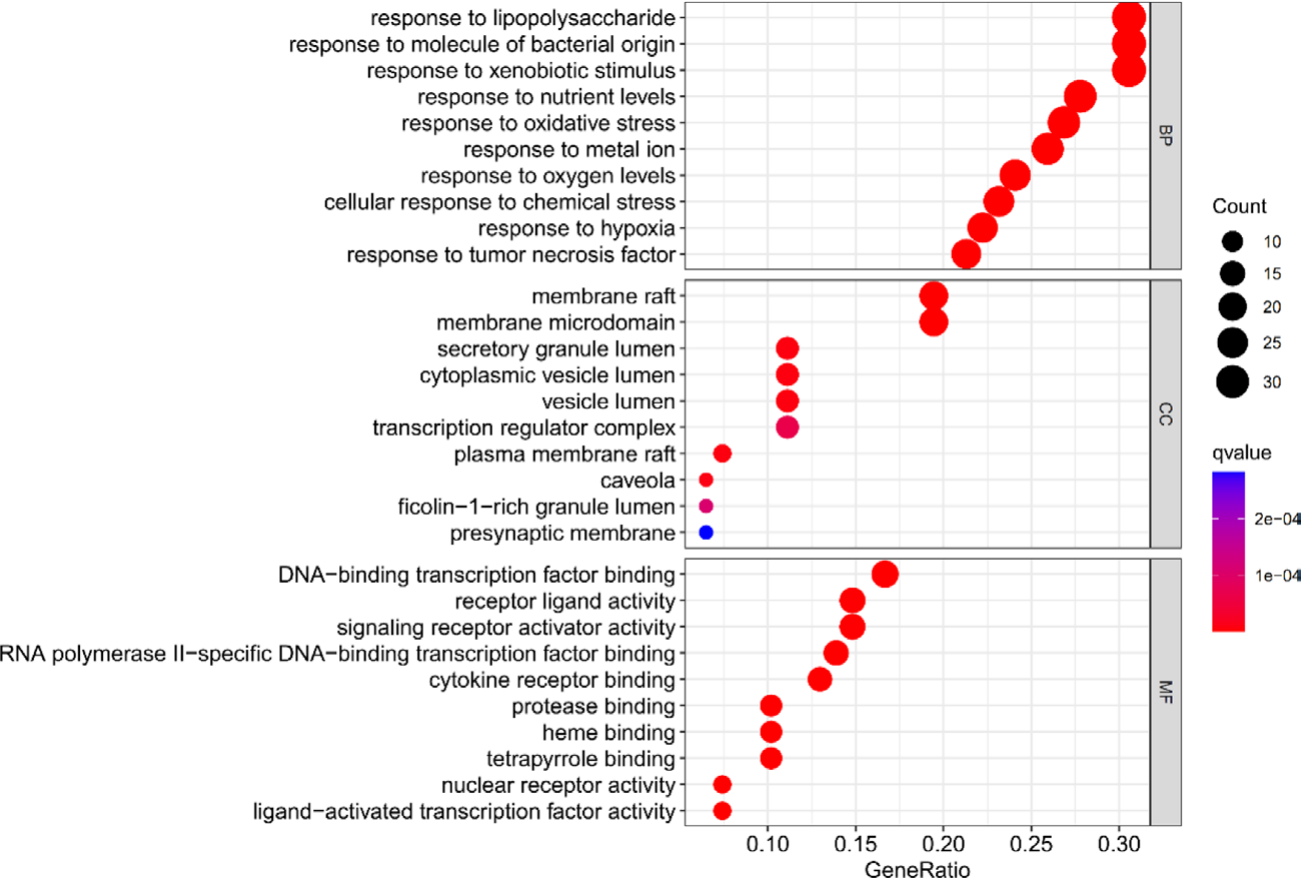
GO function enrichment analysis. GO = gene ontology.

Based on the R packet in Bioconductor, the core target is analyzed by GO function enrichment analysis and KEGG pathway analysis. The enrichment condition is *P* value Cutoff = .05 and *Q* value Cutoff = 0.05; other projects default to the original setting and draw bubble charts and histograms according to the *P*-value, Q value, and the number of genes enriched on each project. The Abscissa represents the number of target points. According to GO functional enrichment analysis, 2304 items were identified, of which 2091 were identified by biological process, 59 by cellular components, and 154 by molecular function. The related items of biological process are related to the responses to lipopolysaccharide, bacterial molecules, nutrient levels, and oxidative stress. The related cellular components items mainly include membrane raft, membrane microdomain, etc. The associated items of molecular function include DNA-binding transcription factor binding, receptor-ligand activity, and signal receptor activator activity (Fig. 5).

**Figure 6.**
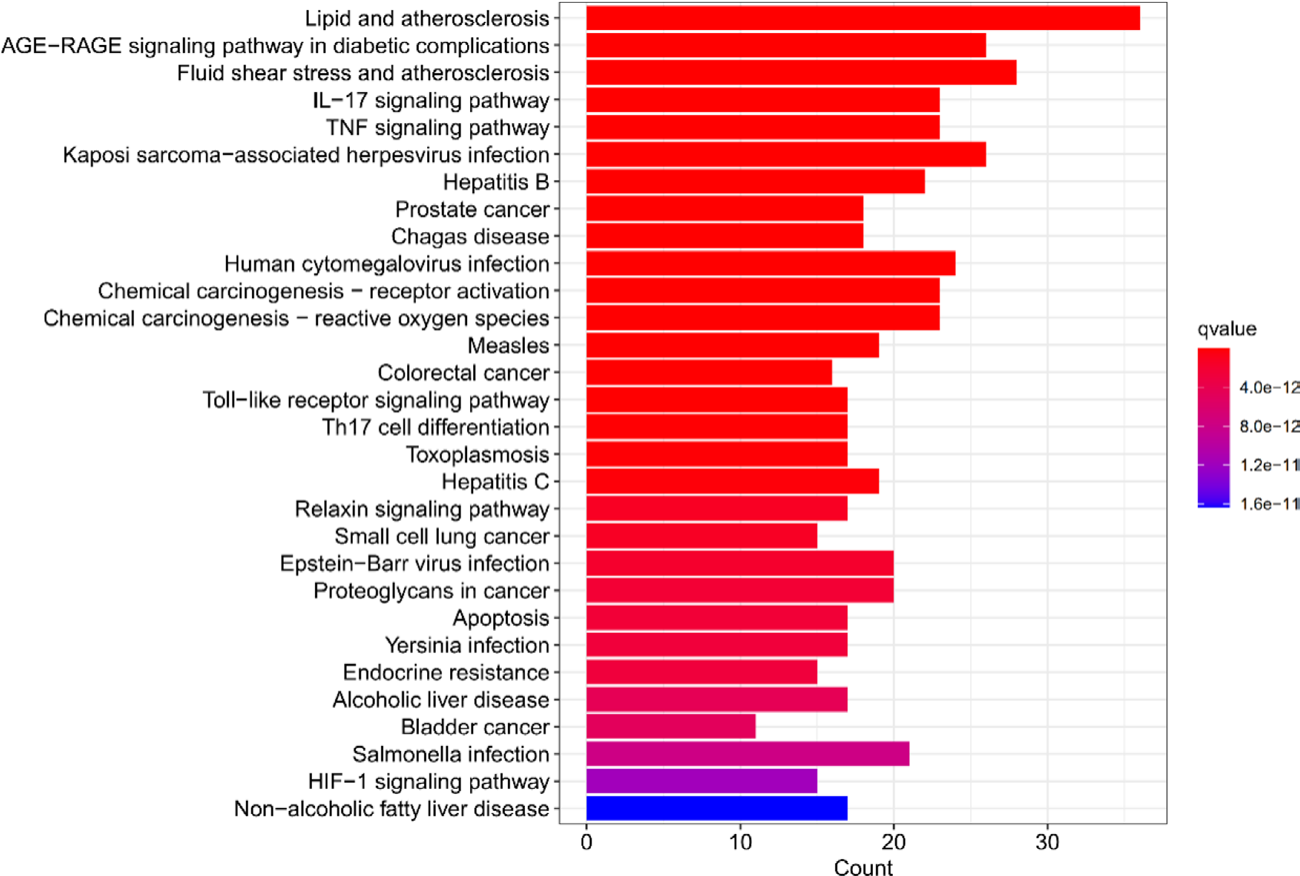
KEGG pathways enrichment analysis. KEGG = Kyoto Encyclopedia of Genes and Genomes.

**Figure 7.**
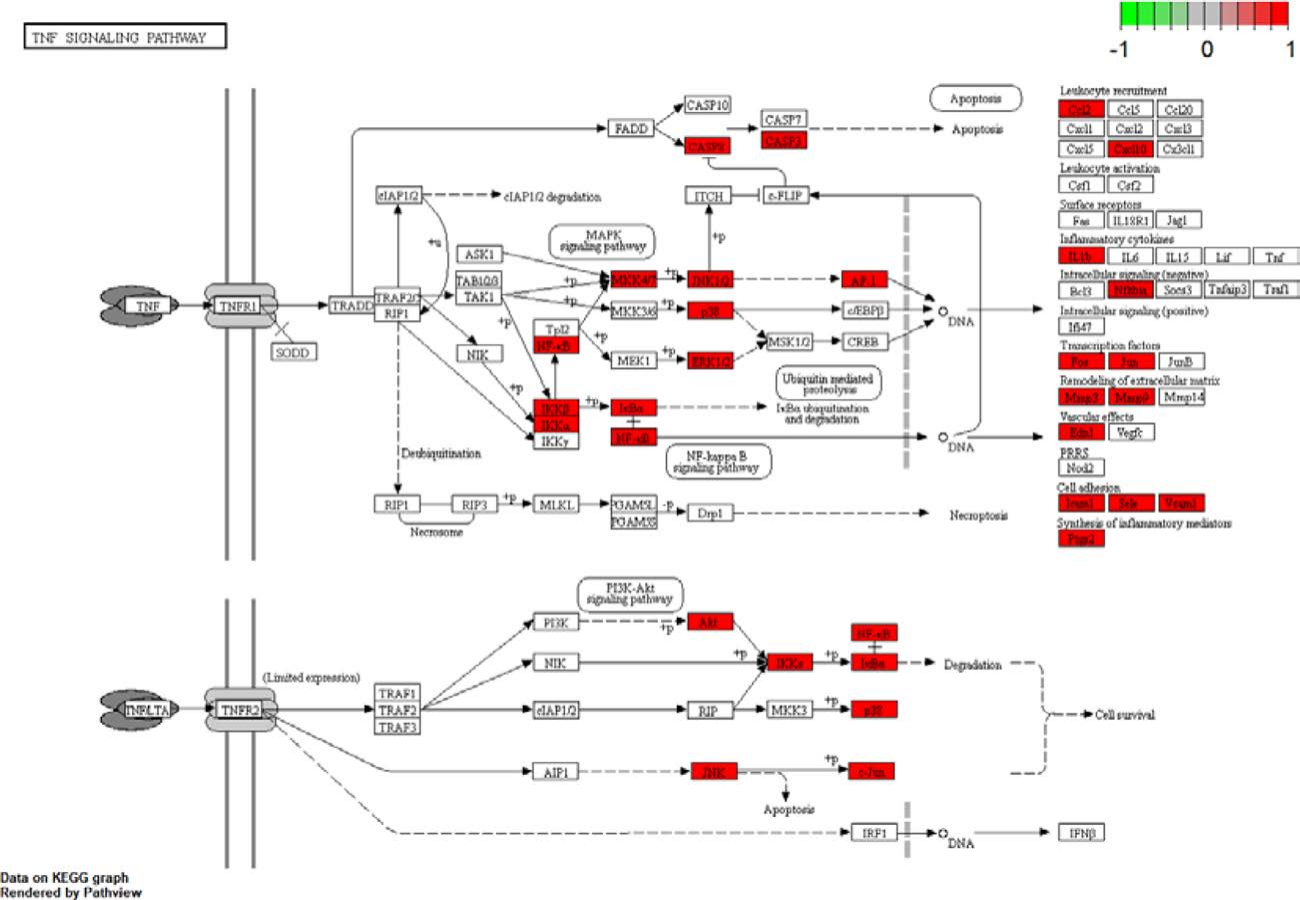
TNF signaling pathway.

The analysis of KEGG pathways produced 155 pathways, from which 30 paths with rich gene information were selected for the histogram. Potential targets of Duhuo Jisheng decoction in OA treatment are mostly the AGE − RAGE signaling pathway, IL − 17 signaling pathway, TNF signaling pathway, and Toll − like receptor signaling pathway (Fig. 6). An example of further visualization can be found in the TNF signaling pathway, which is closely linked to OA (Fig. 7). The marked red node represents the critical target in the pathway. OA with Duhuo Jisheng decoction is thought to be effectively treated by the TNF signaling pathway.

### 3.6. Molecular docking

**Figure 8.**
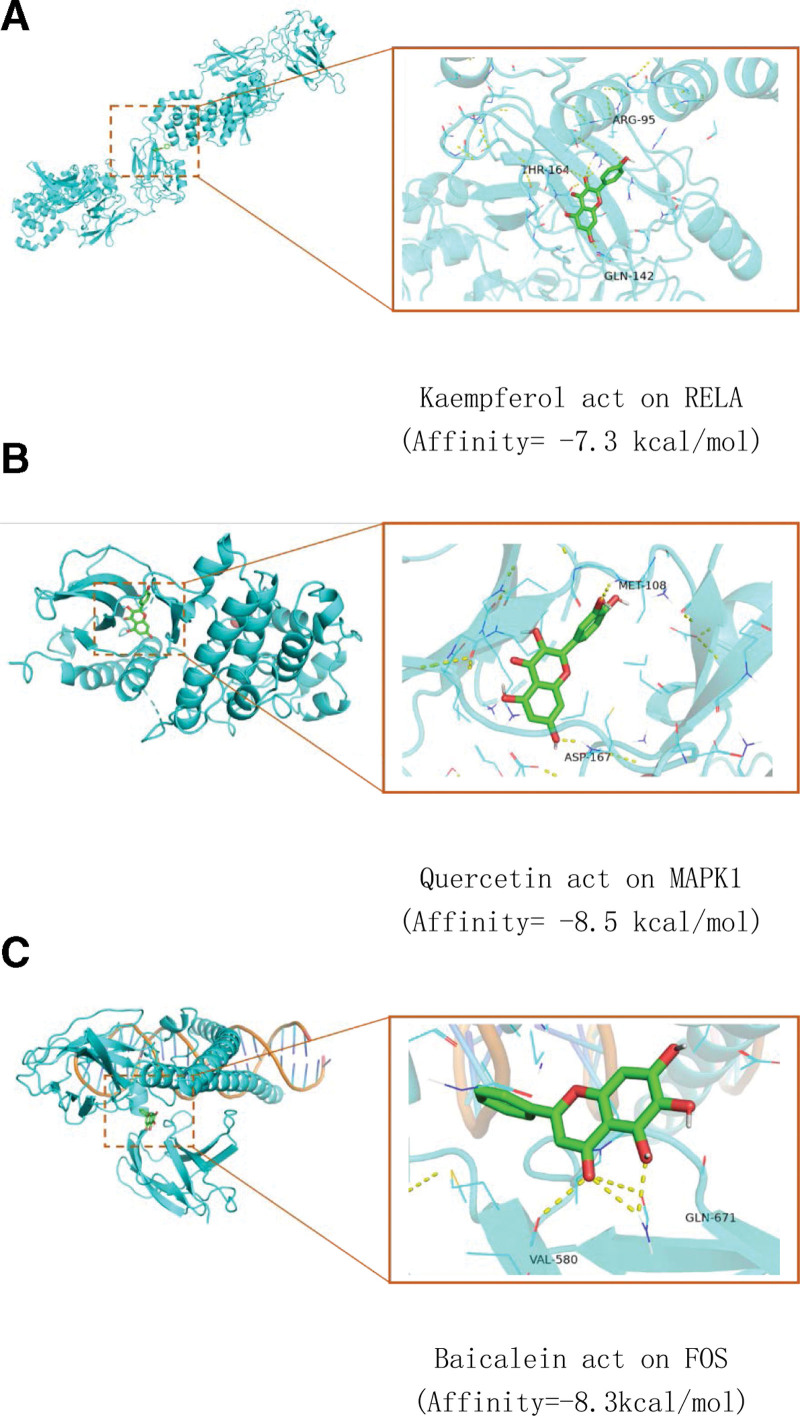
Duhuo Jisheng Decoction-OA core target molecule docking. OA = Osteoarthritis.

The selected core targets are docked with some core components. It is generally believed that the drug molecules with binding energy ≤ −5.0 kJ/mol have good critical activity with the target. We molecularly docked 7 key targets (RELA, FOS, STAT3, MAPK14, MAPK1, JUN, and ESR1) in PPI with their corresponding active components. The details of the 4 optimal molecular docking targets and their associated active components are shown in Figure 8. The results of molecular docking showed that the affinity between the vital functional components and binding targets of Duhuo Jisheng decoction was much less than that of −5.0 kJ/mol, which indicated that the predicted critical active ingredients of Duhuo Jisheng decoction had a good bonding activity with the essential targets for the treatment of OA, which proved that the prediction of this Study was more reliable.

## 4. Discussion

OA belongs to “rheumatism involving bone” in traditional Chinese medicine. From the Warring States Period onward, the Inner Canon of the Huangdi Neijing in Chinese records: The beginning of Dubai disease is also born in the wind, rain, cold and summer, wet and happy. Irritability hurts dirty, wind and rain hurt up, and dampness hurts. It is described that bone arthralgia is caused by 3 external pathogens of wind, cold, and dampness in winter. According to the Huangdi Neijing, it is also recorded that Less blood gas is less bearded, less blood gas is unnecessary, feel cold and wet is good. Bone pain, too. Deficiency of vital qi, external evil invading tendons and bones, forming tendon arthralgia. Evil goes deep into the meridians and collaterals, blocking qi, blood, and essence, unable to nourish tendons and bone joints, and joint activity is stagnant. Liver stores blood in tendons, and the kidney stores marrow in essence. If the liver loses its qi and the kidney loses its essence, it can cause liver blood deficiency, kidney essence loss, and bone loss. If it lasts for a long time, there will be joint contracture, poor flexion, and extension. Modern studies have found that the pathogenesis of OA is mainly related to the increase of pro-inflammatory cytokines, the activation of inflammation-related signal pathways, and the degradation of the extracellular matrix.^[11–13]^ The loss of metabolic homeostasis is caused by the imbalance between anabolic and catabolic pathways in the cartilage at the molecular level.^[14]^ IL-1 β is a pro-inflammatory cytokine and is an effective inducer of many catabolic factors in OA, including matrix degradation of metalloproteinases (MMPs) and aggrecanases.^[15]^ Furthermore, IL-1 β also increases the expression and production of inflammatory mediators that contribute to the pathogenesis of OA.^[16–18]^ Some studies have shown that oxidative stress caused by excessive production of reactive oxygen species (ROS) and reactive nitrogen substances (RNS) is an important component in the multifactorial etiology of OA.^[19,20]^ At present, non-steroidal anti-inflammatory drugs (NSAIDs) are usually used to relieve OA symptoms. Long-term use of NSAIDs will cause adverse reactions.^[21,22]^ On the other hand, Duhuo Jisheng decoction can reduce the index of inflammation and have fewer side effects when it can improve the symptoms such as arthralgia in patients with OA.^[23]^

Duhuo Jisheng decoction is derived from the Bei Ji Qian jin Yao fang (Essential Prescriptions for urgent Needs of a Thousand Gold) written by Sun Simiao, a doctor of the Tang Dynasty. Duhuo Jisheng decoction has the functions of dispelling wind and dampness, stopping bi and pain, supplementating qi and blood, and tonifying liver and kidney. Duhuo has the functions of dispelling wind, removing dampness, dispersing cold and relieving pain. It is an essential medicine for the treatment of rheumatism and pain. Sangjisheng has the functions of dispelling wind and dampness, tonifying liver and kidney, strengthening muscles and bones, and preventing fetus. Duhuo and Sangjisheng are used together to dispel wind and damp, dispel cold and relieve pain, and nourish liver and kidney. Qinjiao and Fangfeng can help Duhuo dispel wind and dedampness, and Niuxi and Duzhong can help Sangjisheng nourich liver and kidney. Therefore, Qinjiao, Fangfeng, Niuxi and Duzhong are the main drugs together. Xixin powder for cold and pain relief, Rougui Wenyang powder for cold, Danggui, Dihuang, Shaoyao nourish blood and promote blood circulation, Renshen, Fuling tonifying qi and invigorating spleen were all adjuvant drugs. Gancao reconcilates all the herbs to make medicines.

Through the network pharmacological analysis of Duhuo Jisheng decoction, it is known that there are 254 active components. The traditional Chinese medicine-active ingredient-action target network shows that quercetin, kaempferol, wogonin, baicalein, and β-carotene can match more targets, which may be the critical components of Duhuo Jisheng decoction in the treatment of OA. Quercetin is a naturally occurring flavonoid proved to has anti-inflammatory, antioxidant, and other pharmacological effects.^[24,25]^ It can slow down the development of degenerative diseases by reducing the expression of IL-1 β and IL-6 pro-inflammatory genes in macrophages and adipocytes induced by chemokines.^[26–29]^ It also showed anti-inflammatory properties in TNF-α-induced retinal pigment epithelial cells by down-regulating the production of ICAM-1 and MMP-9.^[30]^ As with quercetin, kaempferol is structurally similar and has various pharmacological properties, including oxidative stress inhibition, morphological change reduction, apoptosis inhibition, and anti-inflammatory activity.^[31–33]^ Multiple stimuli, including propanol, ethanol, hyperglycemia, and lipopolysaccharide, strongly inhibit apoptosis and inflammation.^[33–36]^ It has been reported that kaempferol has anti-apoptosis and anti-inflammatory effects on rat OA chondrocytes stimulated by IL-1 β.^[37]^ Moreover, kaempferol administration relieved OA symptoms in patients with OA when tested in clinical trials.^[38]^ Other biological properties of wogonin include its anti-inflammatory and anti-cancer properties.^[39,40]^ It was found that the protective effect of wogonin on cartilage was proved by effectively inhibiting MMPs and other catabolic genes in OA chondrocytes.^[41,42]^ Baicalein inhibits the proliferation of synovial cells by inhibiting the proliferation of preosteoblasts and promoting the apoptosis of preosteoblasts to reduce the number of differentiated osteoblasts and damage the angiogenesis of endothelial cells.^[43–45]^ Intraarticular injection of baicalein can improve the remodeling of subchondral bone.^[46]^β-carotene is highly absorbed and utilized in the human body, has the ability of antioxidation, and affects inflammation and metabolic syndrome.^[47–49]^ Adipocytes with metabolic dysfunction are protected from oxidative stress and inflammation by glucocorticoids.^[50–52]^ Therefore, the main active ingredients of Duhuo Jisheng decoction for OA may be quercetin, kaempferol, wogonin, baicalein and β-carotene.

The results of the PPI network analysis show that RELA, FOS, STAT3, MAPK14, MAPK1, JUN, and ESR1 may be the critical target of Duhuo Jisheng decoction in the treatment of OA. OA is mainly caused by chondrocyte apoptosis, inflammatory factors, and related hydrolyzed proteins that destroy chondrocytes, and the above proteins have different relationships with these causes.^[50,53]^RELA, a subunit of the NF-β p65 gene, is not only involved in the expression of genes involved in inflammation but also in cartilage formation and differentiation, cell survival, and catabolic enzyme production.^[54–56]^RELA and STAT3 are critical mediators in the process of OA-related underlying diseases. Their interaction can regulate intracellular inflammation and metabolism and drive inflammatory cytokines and immune responses in the OA microenvironment, which play an essential role in the pathogenesis of OA.^[12,57,58]^ JUN is a subunit of AP-1. Stimulation of JNK can promote JUN phosphorylation and expand inflammatory response.^[59]^ ESR1 is a ligand-dependent transcription factor, often expressed in chondrocytes, osteoclasts, osteoblasts, and bone marrow stromal cells.^[60]^ Estradiol targeting ESR1 can inhibit the ERK signal pathway, activate autophagy, inhibit apoptosis and regulate chondrocyte proliferation.^[61]^

According to the results of GO enrichment analysis, it is concluded that the treatment of OA with Duhuo Jisheng decoction mainly involves biological processes such as lipopolysaccharide, oxidative stress, nutrition level, and so on. Lipopolysaccharide is a standard endotoxin, which can activate mononuclear macrophages, endothelial cells, and epithelial cells through a cellular signal transduction system, synthesize and release various cytokines and inflammatory mediators, and then cause a series of reactions in the body.^[62,63]^ After a series of reactions, the corresponding NF-κ B and MAPK pathways can be activated, resulting in the release of IL-1, IL-6, TNF-α, NO, and so on, resulting in the inflammatory response.^[64,65]^ Lipopolysaccharide stimulates the expression of pro-inflammatory cytokines (including TNF-α) in traditional immune tissues and skeletal muscle.^[66]^ Lipopolysaccharides cause inflammation and are pro-inflammatory compounds derived from bacteria. Notably, the function of lipopolysaccharide or molecules from bacteria provides thermal stability for the virus.^[67]^ Studies have found that oxidative stress is an important mechanism, and oxidant-antioxidant imbalance can lead to pathological effects related to OA, such as joint inflammation, cartilage injury, and synovitis.^[68]^ Reactive oxygen species (ROS) and reactive nitrogen (RNS) are produced under the physiological conditions of the human body. Due to the weakening of the antioxidant defense mechanism and the increase of lipid peroxidation, these reactive oxygen species can lead to oxidative damage to various components of joints.^[20]^

The KEGG pathway enrichment analysis results showed that Duhuo Jisheng decoction mainly involved the AGE − RAGE signaling pathway, IL − 17 signaling pathway, TNF signaling pathway, and Toll − like receptor signaling pathway in the treatment of OA. The classical signal pathway of autophagy in the AGE-RAGE pathway plays an essential role in the homeostasis of articular chondrocytes.^[69]^ It has been found that AGEs bind to RAGE to produce oxidative stress, increase the production of reactive oxygen species (ROS), trigger the activation of intracellular conduction channels such as MAPKs and NF-kB, and induce the expression and release of a large number of pro-inflammatory cytokines (IL-1, IL-6), growth factors (TGF-B, IGF) and adhesion molecules (VCAM-1, ICAM-1).^[70–72]^ The further formation of positive feedback, the continuous activation of cells, and the imbalance of synthesis and catabolism of chondrocytes are fundamental reasons leading to and aggravating the pathological changes of 0A.^[73]^ A toll-like receptor signaling pathway is a pattern recognition receptor. Its activation is closely related to the production of matrix metalloproteinases, proteinase, tumor necrosis factor, transforming growth factor, and IL-1, which are involved in joint destruction. Toll − like receptor signaling pathway may be interested in mediating the joint destruction response of OA.^[73]^In the Study of chondrocyte apoptosis, it is found that inflammatory cytokines play an essential role in the pathogenesis of OA. They participate in many physiological metabolism and functional regulation and maintain the standard tissue structure and function.^[74]^ TNF-a can interact with IL-1 β to activate matrix metalloproteinases, inhibit the synthesis and metabolism of chondrocytes, induce apoptosis of chondrocytes, and finally cause cartilage destruction.^[75]^ With the apoptosis of chondrocytes, some apoptotic products can enhance the expression of IL-1 β and TNF-a, which leads to a vicious circle and accelerates the progress of OA.^[76]^ Members of the IL-17 family can mediate multiple inflammatory disease processes. Previous studies have shown that inhibition of the biological effects of IL-17 and its receptors reduces synovial inflammatory responses in a rat model of rheumatoid arthritis and protects against collagen damage in a mouse model of arthritis, and that modulation of IL-17 gene expression levels leads to cascade enhancement of these biological effects.^[77–79]^ Ji et al^[80]^ showed that in rheumatoid arthritis, IL-17 produces elevated levels of inflammatory cytokines such as TNF-α, IL-1b and IL-6 through spur cells, and specific soluble inhibitors can reduce IL-6 and collagen degeneration-related markers in synovial and bone tissues by blocking the biological effects of IL-17. The occurrence and development of OA are closely related to inflammatory factors, oxidative stress, apoptosis, autophagy, etc. It can be seen that Duhuo Jisheng decoction can interfere with the course of knee OA through multi-target and multi-signal pathways.

Through a network pharmacological analysis, we identified a relationship between OA and early atherosclerosis in obese patients. The mechanical stress in weight-bearing joints caused by obesity stimulates mechanoreceptors and detrusor-activated ion channels in the joints,^[81,82]^ activating inflammatory signaling pathways to produce inflammation,^[83,84]^ while the activation of detrusor-activated ion channels triggers an increase in intracellular calcium ion concentration, leading to mitochondrial DNA damage and disrupting the balance between chondrocytes, extracellular matrix and subchondral bone and the body’s nutrient metabolism, which plays a key role in the triggering and progression of OA and atherosclerosis.^[85]^ However, a higher prevalence of non-weight-bearing OA has been observed in obese patients, and it has been hypothesized that metabolic factors may play an essential role in non-weight-bearing OA.^[86–88]^ Recent data found that mechanical stress in OA produces inflammation through mechanoreceptors present on the surface of chondrocytes,^[89]^ which explains the recent finding that in vitro chondrocyte cultures were subjected to compressive stress that enhanced the expression of IL-1β, IL-6 and COX-2 and the synthesis of PGE2 in fibroblast-like synoviocytes, thus participating in the development of OA.^[90–92]^ Adipose tissue also synthesizes cytokines such as TNF-α and IL-6, and in particular, the infrapatellar fatty pad releases a variety of inflammatory and adipokines involved in OA progression,^[93,94]^ so that chronic inflammation plays a key role in the progression of OA and atherosclerosis and subsequent cardiovascular disease. In fact, obesity as a significant risk factor for OA and atherosclerosis co-morbidity and the characterization of OA as a metabolic syndrome provides a fresh perspective in the understanding of OA itself and its potential treatment.^[95,96]^

To sum up, Duhuo Jisheng decoction in the treatment of OA regulates the development of the disease from multi-components, multi-targets, and multi-pathways, and its active components may exert its curative effect by inhibiting inflammation, regulating apoptosis, and reducing oxidative stress. This paper provides a specific theoretical basis for the clinical application of Duhuo Jisheng decoction and provides a particular direction for follow-up research, but there are some limitations. First, the research result is a virtual prediction result, which needs to be further verified by follow-up experiments. Secondly, the composition of traditional Chinese medicine compounds is complex. The interaction between active components, pharmacokinetics in vivo, and regulation intensity on each target cannot be fully included in the calculation, so the exact mechanism still needs further study. In addition, although a large number of targets and pathways can be screened through network pharmacology, it still needs to be further verified by in vivo and in vitro experiments to clarify the molecular mechanism of Duhuo Jisheng decoction in the treatment of OA, promote the scientific clinical research of traditional Chinese medicine, and speed up the modernization of traditional Chinese medicine. The potential mechanism of Duhuo Jisheng decoction in the treatment of OA was analyzed by network pharmacology and molecular docking technology. The results showed a close synergistic relationship between the active components of Duhuo Jisheng decoction and its action target and good binding activity, which provided a new idea and direction for the future Study of the molecular mechanism of Duhuo Jisheng decoction in the treatment of OA.

## 5. Conclusion

Studies have shown that the mechanism of Duhuo Jisheng decoction in the treatment of OA may be achieved through a multi-component, multi-target, multi-signal pathway. We found that quercetin, kaempferol, wogonin and baicalein, and β-carotene can be identified as 5 crucial active components. The critical therapeutic target may be RELA, FOS, STAT3, MAPK14, MAPK1, JUN, and ESR1. The essential pathways of a signal of Duhuo Jisheng decoction in treating OA are the AGE-RAGE pathway, IL-17 pathway, tumor necrosis factor pathway, and Toll-like receptor pathway. The possible pharmacological mechanism is mainly related to lipopolysaccharide, oxidative stress, and nutritional level. In addition, there are few reports on some active components, target genes, and signal transduction pathways in this study, which may provide clues for the further study of the mechanism of Duhuo Jisheng decoction in the treatment of OA. As a result of our study, we have provided valuable evidence for further basic research and clinical application.

## Acknowledgments

The Natural Science Foundation of Jilin Province (20210101189JC) provided support for this work.

## Author contributions

**Investigation:** Weidong Zhang, Zhenhai Cui.

**Methodology:** Zhenhai Cui, Xuezhen Le.

**Software:** Weidong Zhang, Kunyu Song.

**Validation:** Weidong Zhang, Chunliang Zhang.

**Visualization:** Weidong Zhang, Zhenhai Cui.

**Writing – original draft:** Weidong Zhang, Liquan Sha.

**Writing – review & editing:** Wenhai Zhao, Liquan Sha.
